# Response of the nitrergic system to activation of the neuroendocrine stress axis

**DOI:** 10.3389/fnins.2015.00003

**Published:** 2015-01-21

**Authors:** Hsiao-Jou Cortina Chen, Jereme G. Spiers, Conrad Sernia, Nickolas A. Lavidis

**Affiliations:** School of Biomedical Sciences, The University of QueenslandBrisbane, QLD, Australia

**Keywords:** anxiety, depression, hypothalamic-pituitary-adrenal axis, glucocorticoids, nitrergic system, nitric oxide, peroxynitrite, reactive nitrogen species

## Abstract

Exposure to stressful stimuli causes activation of the hypothalamic-pituitary-adrenal axis which rapidly releases high concentrations of glucocorticoid stress hormones, resulting in increased cellular metabolism and spontaneous oxygen and nitrogen radical formation. High concentrations of nitrogen radicals, including nitric oxide, cause damage to cellular proteins in addition to inhibiting components of the mitochondrial transport chain, leading to cellular energy deficiency. During stress exposure, pharmacological inhibition of nitric oxide production reduces indicators of anxiety- and depressive-like behavior in animal models. Therefore, the purpose of this review is to present an overview of the current literature on stress-evoked changes in the nitrergic system, particularly within neural tissue.

## Introduction

An acute stress response is mediated by the tripartite activation of the sympatho-adrenal-medullary (SAM), hypothalamic-spinal-adrenal (HSA), and hypothalamic-pituitary-adrenal (HPA) axes. The first of these axes to respond is the autonomic SAM system, consisting of several hypothalamic and brainstem nuclei, notably including the locus ceruleus (Jansen et al., 1995). The locus ceruleus is the primary source of central noradrenergic signaling, functioning via the ascending noradrenergic bundle and descending through preganglionic neurons in the intermediolateral cell column (IML) of the spinal cord to innervate the adrenal medulla (Sara, 2009; Ulrich-Lai and Herman, 2009). Through the combined action of catecholamines, this system promotes increased arousal and vigilance and is responsible for the rapid generation of the “fight-or-flight” response (Jansen et al., 1995). The paraventricular nucleus (PVN) of the hypothalamus is considered the apex of the HPA stress response as release of corticotropin-releasing hormone from the parvocellular neurosecretory neurons triggers anterior pituitary corticotrophs to release the pro-opiomelanocortin fragment, adrenocorticotropic hormone (ACTH), into the circulation. However, the PVN also facilitates corticosterone release directly through the HSA stress axis via adrenocortical innervation from the IML, and indirectly via an alternative stress pathway involving prolactin release (Buijs et al., 1999; Lowry, 2002; Ulrich-Lai et al., 2006; Jaroenporn et al., 2009). This ultimately sensitizes the adrenal gland to ACTH, resulting in corticosterone release from the *zona fasciculata* of the adrenal cortex thereby exerting the characteristics downstream cellular and metabolic effects of stress (Buijs et al., 1999; Lowry, 2002; Weiser et al., 2011). Adrenal glucocorticoids accelerate cellular metabolism to increase available energy which consequently increases free radical formation in specific regions of the central nervous system (Spiers et al., 2013). This stress-induced increase in radical production, including nitric oxide (NO) formation, leads to oxidative and nitrosative stress (Chen et al., 2014). Furthermore, the toxic metabolite of NO, peroxynitrite, is capable of inhibiting components of the mitochondrial respiratory chain, leading to cellular energy deficiency (Sarti et al., 2012). Since dysfunction of the nitrergic system has been implicated in the neuropathogenesis of several stress-related disease states, the present review summarizes our current understanding and advances relating to the impact of stress on the nitrergic system.

## Nitric oxide biosynthesis and functions

**Figure 1:**
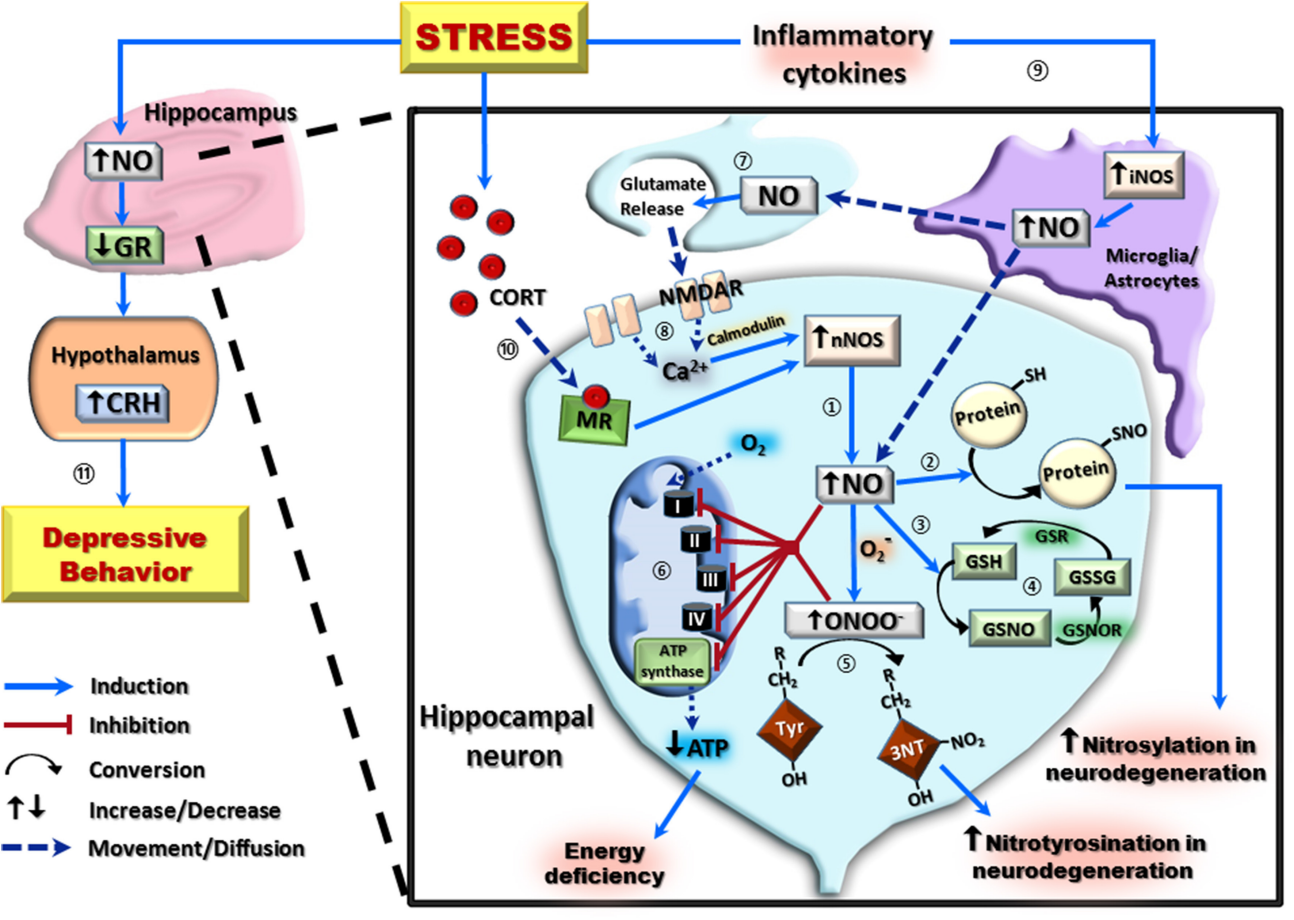
**A schematic representation of the nitrergic system and its downstream effects in hippocampal neurons following stress exposure**. In hippocampal neurons, the majority of nitric oxide (NO) production occurs via the conversion of L-arginine to L-citrulline by the neuronal isoform of nitric oxide synthase (nNOS) ①. High concentrations of NO can then covalently bond with protein thiol groups (protein-SH) to form S-nitroso-proteins (protein-SNO) ② or interact with the reduced form of glutathione (GSH) forming S-nitrosoglutathione (GSNO) ③. This can be regenerated back to GSH via an initial conversion to oxidized glutathione (GSSG) by S-nitrosoglutathione reductase (GSNOR), and subsequent reduction of GSSG by glutathione reductase (GSR) ④. Interaction of NO with the superoxide radical (O_2_^−^) results in the formation of the neurotoxic radical, peroxynitrite (ONOO^−^) which irreversibly reacts with protein tyrosine (Tyr) residues to form 3-nitrotyrosine (3-NT) ⑤. Increased NO and ONOO^−^ are capable of causing cellular energy deficiency by inhibition of all components of the electron transport chain (Complex I–IV), including ATP synthase, resulting in decreased ATP production ⑥. Both post-synaptically produced NO, and NO produced by the inducible isoform of nitric oxide synthase (iNOS), can act as a neurotransmitter on pre-synaptic neurons ⑦. This pre-synaptic NO causes glutamate release, which activates post-synaptic NMDA receptors (NMDAR) to increase calcium (Ca^2+^) concentration and, in the presence of calmodulin, further potentiate nNOS-derived NO ⑧. Stress exposure increases NO by activating inflammatory cytokines to potentiate glial/astrocyte iNOS activity ⑨, and by increasing circulating corticosterone (CORT) which induces nNOS activity via a mineralocorticoid receptor (MR)-mediated pathway ⑩. This increase in NO results in downregulation of hippocampal glucocorticoid receptors (GR) and subsequently increases hypothalamic corticotropin-releasing hormone (CRH) to induce depressive-like behaviors ⑪.

Nitric oxide, a gaseous free radical belonging to the family of reactive nitrogen species (RNS), is synthesized through the conversion of L-arginine to L-citrulline by nitric oxide synthase (NOS) in the presence of oxygen, NADPH, and cofactors such as tetrahydrobiopterin (Andrew and Mayer, 1999). There are three main isoforms, each with a specific distribution profile; neuronal NOS (nNOS, type I), inducible NOS (iNOS, type II), and endothelial NOS (eNOS, type III) (Stuehr, 1999). Though nNOS is predominantly active in the cytosol of central and peripheral neurons for signaling and regulation, it has also been found in the sarcolemma and cytoplasm of all muscle fibers (Frandsen et al., 1996). Interestingly, nNOS is present in the hippocampus, hypothalamus, pituitary, and adrenal gland, suggesting co-localization with the HPA axis (Lai et al., 2005; Gadek-Michalska et al., 2012). Furthermore, several studies have demonstrated transcriptional regulation of nNOS by glucocorticoids in the hippocampus, implicating its importance in the stress response, although the upstream promoter of NOS1 does not carry a glucocorticoid responsive element (López-Figueroa et al., 1998; Reagan et al., 1999; Zhou et al., 2011). There are four nNOS splice variants, α, β, γ, and μ, with nNOSα being the most dominant and therefore being physically and functionally coupled to the glutamate receptors of the N-methyl-D-aspartate (NMDA) subtype through their mutual post-synaptic density-95/discs-large/zona occludens-1 (PDZ) binding motif (Eliasson et al., 1997). Within the hippocampus, local calcium influx through NMDA receptors can trigger the production of NO, which subsequently activates its receptor, soluble guanylyl cyclase, leading to release of second messenger cyclic guanosine monophosphate (cGMP) (Figure 1). This NO-cGMP signaling has been implicated in the induction of hippocampal long-term potentiation which is known to be one of the principal mechanisms in learning and memory (Schuman and Madison, 1991; Arancio et al., 1996; Kelley et al., 2010). The nNOSμ mainly localizes in the skeletal muscles, with nNOSμ-deficient muscles being myopathic (Percival et al., 2008). The β variant lacks the PDZ domain while nNOSγ has very little to no enzymatic activity (Eliasson et al., 1997). Endothelial NOS contains a putative shear stress responsive element in the promoter region of the NOS3 gene while the protein is membrane-bound to the golgi apparatus and caveolae, producing NO mainly in the endothelium of blood vessels responsible for vasodilation and smooth muscle relaxation (Smith et al., 2006). The inducible form of NOS responds at the transcriptional level to inflammatory factors (Zamora et al., 2000; Aktan, 2004). Within the central nervous system, the iNOS-mediated release of NO by astrocytes and microglia has a major role in antimicrobial and tumoricidal activity in response to various inflammatory signals (Hua et al., 2002; Brantley et al., 2010). Moreover, upon transcriptional activation, this soluble subtype can produce micromolar levels of NO and is known to be associated with diseases such as artherosclerosis, rheumatoid arthritis, diabetes, septic shock, and multiple sclerosis (Kuhlencordt et al., 2001; Hill et al., 2004; Maki-Petaja et al., 2008; Heemskerk et al., 2009; Soskic et al., 2011). Both nNOS and eNOS are constitutively active isoforms producing low concentrations of NO (in the nanomolar range) over long periods and are activated by calcium ions though transient binding to the calcium-binding protein, calmodulin (Knott and Bossy-Wetzel, 2009). Comparatively, the inducible form of NOS can produce high concentrations of NO in relatively short periods and is calcium independent due to a high binding affinity to calmodulin (Aktan, 2004). The inorganic ions, nitrate and nitrite (NO*x*), were previously thought to be the end products of NO metabolism. However, recent studies have demonstrated a NOS-independent pathway in which NO can be produced by reducing NO*x*, a reaction catalyzed by xanthine reductase under low oxygen tension and low pH environment. The NO produced by this nitrate-nitrite-NO pathway may have similar roles to NO generated from the L-arginine-NOS pathway representing an important secondary pool (see review by Lundberg et al., 2008).

## Nitrosative stress

High levels of NO and its derivatives are destructive to cellular components such as proteins, lipids and DNA. Nitric oxide can react directly with molecular oxygen to produce two relatively strong oxidants, nitrogen dioxide and dinitrogen trioxide. However, at physiological levels of NO these reactions are relatively slow. A primary reaction in the production of RNS is the combination of NO and superoxide anions to form the highly reactive metabolite, peroxynitrite, a potent neurotoxin (Lipton et al., 1993). It has been suggested that NO and peroxynitrite can disrupt adenosine 5′-triphosphate (ATP) synthase and almost all components of the mitochondrial respiratory chain (Almeida and Bolanos, 2001; Sarti et al., 2012). These RNS reversibly or irreversibly inhibit mitochondrial oxygen consumption, particularly at complex IV (also known as cytochrome *c* oxidase), and may lead to cellular energy deficiency and ultimately cell death in pathological conditions (Sarti et al., 2012). Inhibition of cytochrome *c* oxidase by NO and peroxynitrite causes neuronal dysfunction and, in addition to high iNOS expression, has been observed in the cortex of Alzheimer's patients (Mutisya et al., 1994; Haas et al., 2002).

*S*-nitrosylation is the covalent attachment of NO to the thiol side chain of the amino acid cysteine, forming other NO derivatives termed *S*-nitroso-proteins. Under physiological conditions, it has been demonstrated that NO is converted to the nitrosonium ion which subsequently S-nitrosylates the NMDA receptor, thereby preventing glutamate excitotoxicity by blocking calcium influx, promoting cell survival (Lipton and Stamler, 1994). Excessive production of NO can be counteracted by conjugation with reduced glutathione, forming the stable adduct *S*-nitrosoglutathione which has important role in signal transduction and regulation of a variety of protein functions (Klatt and Lamas, 2000; Anand and Stamler, 2012). Abnormal *S*-nitrosylation to proteins such as apolipoprotein E, cyclin-dependent kinase 5, dynamin-related protein 1, parkin, peroxiredoxin 2, protein disulfide isomerase, heat-shock protein 90, and X-linked inhibitor of apoptosis have all being linked to neurodegenerative conditions such as Alzheimer's and Parkinson's diseases (Anand and Stamler, 2012). Lastly, peroxynitrite provokes protein nitrotyrosination, an irreversible chemical addition of a nitro group to the tyrosine residue in target proteins generating 3-nitrotyrosine. This post-translational modification usually impairs the normal physiological function of the proteins and therefore nitrotyrosination has been used as a marker in several neurodegenerative conditions such as amyotrophic lateral sclerosis (Peluffo et al., 2004). These aspects of the nitrergic system have been summarized in Figure 1.

## Stress-evoked modulation of the nitrergic system

It has been generally accepted that psychophysiological stress is associated with upregulation of NOS mRNA expression and enzymatic activity. For example, a single 6 h acute immobilization stress induces upregulation of iNOS expression and activity in the cerebral cortex which is mediated by the NMDA receptor and subsequent activation of the transcriptional factor, nuclear factor kappa-light-chain-enhancer of activated B cells (NF-κB) (Madrigal et al., 2001). The acute stress-induced activation of the NMDA receptor also increases tumor necrosis factor-alpha (TNFα) via upregulation of TNFα-convertase. Antagonism of TNFα-convertase prevents the stress-induced translocation of NF-κB and subsequent iNOS expression, thus confirming the involvement of TNFα (Madrigal et al., 2002). This is also supported by Shirakawa et al. (2004) who demonstrated glutamatergic activation and not catecholaminergic drive of the hypothalamic paraventricular nucleus to be responsible for the acute stress-induced increase in NO metabolites. Interestingly, biting activity is capable of suppressing the stress-induced increase in hypothalamic nNOS mRNA expression in rats (Hori et al., 2005). A single 2 h acute restraint stress significantly increases the density of neurons expressing nNOS visualized by nicotinamide adenine dinucleotide phosphate-diaphorase (NADPH-d) histochemistry in the amygdaloid nucleus, an effect delayed by 5 days in the hippocampus and entorhinal cortex (Echeverry et al., 2004). Predator-induced post-traumatic stress significantly increases nNOS positive neurons and total NO*x* in the medial prefrontal cortex 7 days after the 10 min predator stress treatment (Campos et al., 2013). Conversely, Chakraborti et al. (2014) demonstrated that acute restraint stress causes a reduction in total NO*x* and an increase in the major endogenous NOS inhibitor, asymmetric dimethylarginine, in whole brain homogenates. This suggests that the stress-induced NO*x* increases in regions such as the hippocampus and hypothalamus may hold a high degree of functional significance. These biochemical changes in NO*x* and asymmetric dimethylarginine were observed alongside anxiety-like behavior and were more pronounced in male compared to female rats. The pharmacological blockade of estrogen biosynthesis exacerbated these biochemical and behavioral changes in females, suggesting that the observed sex differences are due to a protective role of estrogen. Interestingly, bilateral injection of an NMDA receptor antagonist, NOS inhibitor, or NO scavenger into the dorsal hippocampus attenuated autonomic responses such as hypertension and tachycardia following a 60 min acute restraint stress, suggesting that NMDA/NOS activation within the hippocampus plays a role in autonomic modulation during stress (Moraes-Neto et al., 2014). Another study from the same group proposed a glutamatergic NMDA receptor-NO-cGMP signaling pathway in modulating contextual fear conditioning within the dorsal hippocampus, where intra-hippocampal injection of NMDA receptor antagonist DL-AP7, NO scavenger 2-(4-carboxyphenyl)-4,4,5,5-tetramethylimidazoline-1-oxyl-3-oxide (CPTIO), and cGMP inhibitor 1H-[1,2,4]oxadiazolo[4,3-a]quinoxalin-1-one (ODQ), attenuated the fear-conditioned response (Fabri et al., 2014).

Chronic immobilization stress has been shown to increase NO*x*, iNOS activity, and peroxynitrite-induced 3-nitrotyrosine accumulation in cortical neurons (Olivenza et al., 2000). Notably, de Pablos et al. (2014) recently found a degree of regional specificity associated with this chronic stress-induced iNOS expression, with little to no constitutive expression in the substantia nigra following 9 days of unpredictable stress exposure. However, this same unpredictable stress model potentiates iNOS expression following exposure to exogenous immunostimulatory stressors such as lipopolysaccharides. Recent studies in several animal paradigms have demonstrated that inhibitors of NOS significantly modulate stress-related behaviors. In support of these findings, the commercially available antidepressant paroxetine, a selective serotonin reuptake inhibitor, also possesses NOS inhibition capability (Finkel et al., 1996). Wegener and Volke (2010) have reviewed and summarized these studies including data on each of the NOS inhibitor's specificity and potency, and their anxiolytic- and antidepressant-like properties. Chronic unpredictable mild stress increases plasma nitrite levels and iNOS mRNA expression in the cortex, in addition to damaging cortical neurons and inducing depressive-like behavior (Wang et al., 2008; Peng et al., 2012). These effects can be attenuated or prevented using NOS inhibitors, which was demonstrated by intra-hippocampal injection of the selective iNOS inhibitor, aminoguanidine, resulting in suppression of the chronic unpredictable mild stress-induced depressive-like behavior in rats (Wang et al., 2008). Regional infusion of a selective nNOS inhibitor 7-nitroindazole (7-NI) into the hippocampus showed antidepressant-like effects similar to those with the iNOS inhibitor, aminoguanidine (Joca and Guimaraes, 2006). Likewise, the anxiogenic-like behavior observed in rats during ethanol withdrawal is inhibited by administration of the selective iNOS inhibitor, 1400W, into the dorsolateral periaqueductal gray (Bonassoli et al., 2013). The data with intra-cerebral NOS inhibition is further supported by studies using systemic treatment. Intraperitoneal injection of 1400W increases survival of cortical neurons and decreases the depressive-like behavior in mice (Peng et al., 2012). The nNOS inhibitor 1-(-2-trifluoromethylphenyl)-imidazole (TRIM) given systemically 30 min prior to testing induces anxiolytic-like behavior shown by increased time spent in the light compartment of a light-dark compartment test (Volke et al., 2003). Furthermore, TRIM administration decreased the immobility time in the forced swimming test, demonstrating an antidepressant-like effect comparable to the tricyclic antidepressant imipramine. In agreement with these observations, Ulak et al. (2008) injected TRIM intraperitoneally 50 min before a forced swim test and showed the involvement of the serotonergic system in the antidepressant-like actions of TRIM. This was further clarified in a later study in which the serotonin type II receptors were found to be responsible for this effect (Ulak et al., 2010). Furthermore, Joung et al. (2012) demonstrated that following a 2 h immobilization stress, the selective inhibitor 7-NI produced its anxiolytic-like effects shown by an increase in the time spent on the open arms of the elevated plus-maze through the direct reduction of NO metabolites in the PVN and locus ceruleus. A less specific NOS inhibitor, L-N^G^-Nitroarginine methyl ester (L-NAME), injected systemically 30 min prior to testing shows protective effects against chronic swim stress-induced impairment of passive avoidance learning and hyperalgesia in rats (Nazeri et al., 2014). In a similar vein, Ferreira et al. (2012) performed behavioral, genomic, and proteomic analyses in rats and suggested that the antidepressant-like effects of NOS inhibition may involve the expression of additional factors including members of the glutathione redox system.

Genetic animal models have also contributed to the current understanding of nitrergic changes in stress. Thus, inhibition of NO production by nNOS gene deletion in mice suppressed hippocampal neurogenesis and exhibited antidepressant-like properties while nNOS over-expression in the hippocampus was essential for chronic stress-induced depression (Zhou et al., 2007). Recently, a number of studies have proposed a regulatory role of NO on the limbic HPA stress axis. Zhang et al. (2010) used mice lacking the nNOS gene to demonstrate an anxiolytic-like phenotype when tested using an elevated plus-maze, similar to normal mice treated with intra-hippocampal microinjection of the selective nNOS inhibitor 7-NI. The authors proposed a signaling pathway involving the activation of serotonin type IA receptors which mediate, via an unknown mechanism, the downregulation of hippocampal nNOS, leading to a decrease in NO and subsequent inhibition of cAMP response element-binding (CREB) protein phosphorylation. A follow up study elucidated further the link between NO and the HPA axis by showing that chronic mild stress and glucocorticoid exposure lead to hippocampal nNOS overexpression via activating hippocampal mineralocorticoid receptor (MR) (Zhou et al., 2011). The excessive nNOS-derived NO significantly downregulated local glucocorticoid receptor (GR) expression through either the soluble guanylyl cyclase/cGMP or peroxynitrite/extracellular signal-regulated kinase (ERK) signaling pathways. The significant downregulation of GR in the hippocampus leads to an elevation in hypothalamic corticotropin-releasing hormone and the depressive-like behaviors in mice as illustrated in Figure 1. It is important to note that nNOS deletion, infusion of intrahippocampal nNOS inhibitor, and NO-cGMP signaling blockade prevented the chronic mild stress-evoked behavioral modification. Interestingly, this chronic glucocorticoid-induced MR-nNOS-NO pathway is exclusive to the MR-rich hippocampus and drives HPA axis hyperactivity through impaired negative feedback (Zhu et al., 2014).

The considerable body of evidence from animal models is progressively expanding and supported by modest but significant clinical studies. Several reports have shown that increased levels of NO metabolites are present in depressed and autistic patients (Suzuki et al., 2001; Sogut et al., 2003; Lee et al., 2006). Patients with recurrent depressive behavior displayed higher plasma NO*x* concentrations which were associated with cognitive impairment (Talarowska et al., 2012). Galecki et al. (2010, 2011) discovered single nucleotide polymorphisms in exon 22 of the NOS2A gene (iNOS) and exon 29 of the NOSI gene (nNOS) in depressed Caucasian individuals. Furthermore, three single nucleotide polymorphisms located at the regulatory region of NOSI gene are responsible for the susceptibility of an individual to depressive disorders (Sarginson et al., 2014).

## Summary

A growing body of evidence suggests that the etiology of anxiety and depression-related conditions can be derived from the sensitization of particular stress-related circuits that are “primed” following exposure to a short-term stressor. The duration for stress-related circuitry priming far exceeds responses to adrenergic and glucocorticoid-mediated stress responses. Understanding the mechanisms underlying the induction of this long latency will provide a significant link between stress and the pathogenesis of anxiety and depressive disorders. The nitrergic system has been implicated in regulating both short and long-term activation of the stress response, with a variety of NOS inhibitors demonstrating potent anxiolytic and antidepressant activity. The intrinsic cross talk between neuroendocrine stress and nitrergic system activation is now an important physiological consideration. Further understanding the role of this system is important in identifying early players in stress-induced pathological conditions.

## Author contributions

Author Hsiao-Jou Cortina Chen managed the literature searches, wrote the first draft of the manuscript, and produced the graphic. Author Jereme G. Spiers, Conrad Sernia and Nickolas A. Lavidis critically revised the manuscript. All authors have approved the final version of the manuscript for journal submission.

### Conflict of interest statement

The authors declare that the research was conducted in the absence of any commercial or financial relationships that could be construed as a potential conflict of interest.
